# Homeobox Transcription Factors Are Required for Conidiation and Appressorium Development in the Rice Blast Fungus *Magnaporthe oryzae*

**DOI:** 10.1371/journal.pgen.1000757

**Published:** 2009-12-04

**Authors:** Seryun Kim, Sook-Young Park, Kyoung Su Kim, Hee-Sool Rho, Myoung-Hwan Chi, Jaehyuk Choi, Jongsun Park, Sunghyung Kong, Jaejin Park, Jaeduk Goh, Yong-Hwan Lee

**Affiliations:** Department of Agricultural Biotechnology, Center for Fungal Genetic Resources and Center for Fungal Pathogenesis, Seoul National University, Seoul, Korea; The University of North Carolina at Chapel Hill, United States of America

## Abstract

The appropriate development of conidia and appressoria is critical in the disease cycle of many fungal pathogens, including *Magnaporthe oryzae*. A total of eight genes (*MoHOX1* to *MoHOX8*) encoding putative homeobox transcription factors (TFs) were identified from the *M. oryzae* genome. Knockout mutants for each *MoHOX* gene were obtained via homology-dependent gene replacement. Two mutants, *ΔMohox3* and *ΔMohox5*, exhibited no difference to wild-type in growth, conidiation, conidium size, conidial germination, appressorium formation, and pathogenicity. However, the *ΔMohox1* showed a dramatic reduction in hyphal growth and increase in melanin pigmentation, compared to those in wild-type. *ΔMohox4* and *ΔMohox6* showed significantly reduced conidium size and hyphal growth, respectively. *ΔMohox8* formed normal appressoria, but failed in pathogenicity, probably due to defects in the development of penetration peg and invasive growth. It is most notable that asexual reproduction was completely abolished in *ΔMohox2*, in which no conidia formed. *ΔMohox2* was still pathogenic through hypha-driven appressoria in a manner similar to that of the wild-type. However, *ΔMohox7* was unable to form appressoria either on conidial germ tubes, or at hyphal tips, being non-pathogenic. These factors indicate that *M. oryzae* is able to cause foliar disease via hyphal appressorium-mediated penetration, and *MoHOX7* is mutually required to drive appressorium formation from hyphae and germ tubes. Transcriptional analyses suggest that the functioning of *M. oryzae* homeobox TFs is mediated through the regulation of gene expression and is affected by cAMP and Ca^2+^ signaling and/or MAPK pathways. The divergent roles of this gene set may help reveal how the genome and regulatory pathways evolved within the rice blast pathogen and close relatives.

## Author Summary

Pathogens have evolved diverse strategies to cause disease. *Magnaporthe oryzae* is the fungal phytopathogen that causes rice blast and is considered an important model for understanding mechanisms in fungal development and pathogenicity. Asexual reproduction and infection-related development play key roles in *M. oryzae* disease development. The conidium of *M. oryzae* differentiates a specialized structure, an appressorium. The appressorium generates turgor pressure that allows penetration through the mechanical rupture of host cuticle layers. After colonizing host cells, the fungus produces massive conidia via conidiogenesis, serving as secondary propagules for the polycyclic disease. To elucidate molecular mechanisms in asexual reproduction and appressorium-mediated disease development, we identified eight homeobox transcription factors through a genome-wide *in silico* analysis. Characterization using deletion mutants revealed that each homeobox TF functions as a stage-specific regulator for conidial shape, hyphal growth, conidiation, appressorium development, and invasive growth during *M. oryzae* development. Notably, conidiation and appressorium development were entirely abolished in *ΔMohox2* and *ΔMohox7*, respectively. This study also provides evidence that *M. oryzae* is able to cause rice blast by means of hypha-driven appressoria upon responses to host signaling factors. This study will aid in the understanding of regulatory networks associated with fungal development and pathogenicity.

## Introduction

*Magnaporthe oryzae* is an ascomycete fungus and the causal agent of rice blast, the most destructive disease of rice worldwide. The annual yield loss from rice blast is equivalent to rice that could feed about 60 million people [1]. Rice blast has served as an important model for studying molecular plant-pathogen interactions because of its economic significance and genetic tractability of the host and pathogen. More recently, the availability of the genome sequences of both rice and the fungal pathogen has provided a new platform to understand molecular pathogenesis at the genome level [2]–[4].

Like most fungal pathogens, conidia (asexual spores) of *M. oryzae* play a central role in the disease cycle. The conidia attach to the surface of host plants upon hydration and produce germ tubes. This fungus develops a specialized infection structure, an appressorium, at the tip of the germ tube. The appressorium generates enormous turgor pressure (>8 MPa) by accumulating osmolytes including glycerol for penetration through the mechanical rupture of host cell barriers [5]. After penetration, the fungus develops invasive hyphae, colonizes host cells, and produces massive conidia via conidiogenesis, serving as secondary inocula for new infections. This infection cycle may occur many times during the growing season, resulting in explosive disease development. Therefore, understanding the molecular mechanisms involved in conidiation and appressorium development is a prerequisite to provide novel strategies for disease management.

During the last couple of decades, considerable progress has been made in understanding signaling pathways that regulate the infection-related development of this fungus. These include the mitogen-activated protein kinase (MAPK) signaling cascade [6],[7], signaling pathways dependent on secondary messengers such as Ca^2+^ and cAMP [8]–[13], and G-protein-mediated signaling pathways [14],[15]. For example, deletion of genes involved in cAMP and Ca^2+^ signaling pathways has revealed that both are required not only for infection-related fungal development but also for pathogenicity [16],[17]. Disruption of Gα subunits and the MAP kinase gene also indicated the involvement of G-protein and MAP kinase signaling in vegetative growth, sexual reproduction, and pathogenicity in *M. oryzae* [15]. Most studies have focused on well-known upstream signaling pathways, but relatively little information is available on the downstream regulators of appressorium development.

Conidiogenesis is a complex process that involves a cascade of morphological events. *M. oryzae* produces three-celled conidia on a conidiophore, a specialized structure elongated through apical extension of an aerial hypha. Unlike vegetative hyphae, conidiophores rarely branch and their growth is modestly determinate. Several conidia, mostly three to five are arrayed at the tip of a conidiophore in a sympodial pattern after the occurrence of rounds of mitosis. The molecular biology of conidiation has been characterized in detail for *Aspergillus nidulans* and *Neurospora crassa* [18],[19]. The transcription factor (TF) genes *brlA*, *abaA*, and *wetA* are key regulators in the central regulatory pathway of *A. nidulans* conidiation, which coordinately regulates the order of gene activation during conidiophore development and spore maturation. Several other genes, *FlbB*, *FlbC*, *FlbD*, and *FlbE*, act as early regulators that activate the central regulatory pathway in response to the product of FluG activity [19]. In *N. crassa*, conidium-specific *con* genes have been described [20]. The *fl* gene, which encodes a TF, and genes (*frq*, *wc-1*, and *wc-2*) that act in the *Neurospora* circadian clock regulate the morphological transition from filamentous growth to conidiation [18],[21].

Relatively little information exists on conidiation in *M. oryzae* despite its central role in the disease cycle. Deciphering the conidiation pathways may reveal key determinants of initiation and the progress of conidiation, which may provide potential targets for disease control. A few genes are involved in conidial morphology in *M. oryzae*. Mutations at the *SMO* locus cause abnormally shaped conidia [22]. A mutation of the *Acropetal* gene causes head-to-tail arrays of conidia [23]. Deletion of the *CON7* gene encoding a zinc finger TF also causes abnormal conidia with less septa and/or protuberances at the basal scar [24]–[26]. In addition to genes involved in conidial ontology, a recent study showed that a zinc finger TF-coding gene, named *COS1* for conidiophore stalk-less1, is indispensible for an early stage of conidiophore development [27].

Transcriptional regulation is a major mechanism by which alterations in the expression of specific subsets of genes determine development and differentiation in cells. DNA-binding TFs play a pivotal role in the transcriptional regulation of specific target genes necessary for such processes in response to physiological or environmental stimuli. A comparative genome-wide analysis revealed that a variety of TFs are abundantly present in metazoans, including fungi [28]. *M. oryzae* appears to possess over 400 TF genes, but only a few have been characterized [10],[24],[27],[29],[30]. Homeobox TF genes contain highly conserved sequences coding for the DNA-binding motif called the homeodomain. This group of homeobox TFs was first discovered in *Drosophila melanogaster* in which they specify the body plan along the antero-posterior axis [31],[32]. Numerous genes for homeobox TFs have since been identified in eukaryotes, including fungi. Several studies have established the involvement of homeodomain proteins in mating and sexual differentiation in fungi [33]–[37]. A gene encoding a homeodomain protein also controls hyphal morphology and conidiogenesis in *Podospora anserina* [38]. In *Ustilago maydis*, homeobox TFs regulate the hyphal growth, pathogenicity and sexual cycle [39]. It is therefore evident that homeobox TFs play important regulatory roles in morphogenesis and pathogenesis in plant-pathogenic fungi.

As a first step in deciphering the biological functions of TF genes in *M. oryzae*, we comprehensively searched currently available sequences at the genome-wide level for the existence of homeobox TFs in the fungal kingdom. This analysis revealed that a total of eight genes encoding putative homeobox TFs exist in *M. oryzae*, which were here named *MoHOX1* to *MoHOX8*. Similarly, other fungal species appear to possess multiple copies of these genes. To further characterize the roles of *MoHOXs* in *M. oryzae* biology, deletion mutants of each *MoHOX* gene were generated through a homology-dependant gene replacement strategy. Analyses of the various transformants including *ΔMohox* mutants demonstrated that homeobox TFs function as stage-specific regulators in fungal development and pathogenicity in *M. oryzae*. In particular, *MoHOX2* and *MoHOX7* are indispensable for conidiation and appressorium development, respectively. Our findings would provide new insight into the transcriptional regulation of infection-related morphogenesis at the genome level.

## Results

### Phylogenetic analysis of fungal homeobox TFs

**Figure 1:**
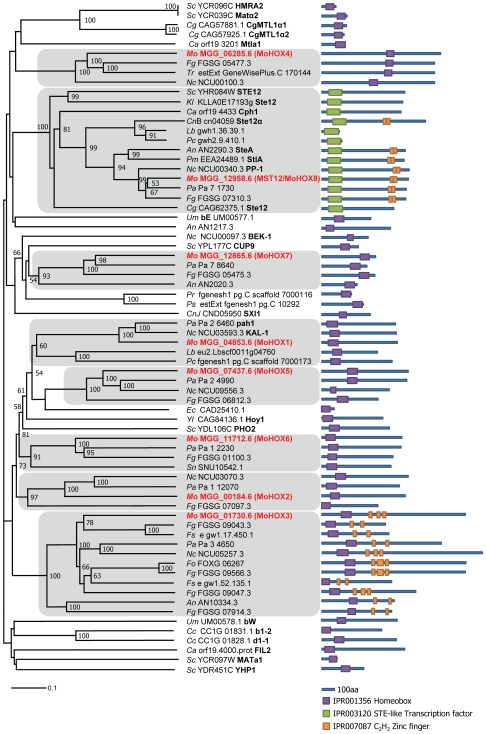
Phylogenetic analysis of putative homeobox transcription factors in fungi. A neighbor-joining tree was constructed based on the amino acid sequences of representative fungal homeodomain TFs. The numbers at nodes represent the percentage of their occurrence in 10,000 bootstrap replicates; only nodes supported by 50 bootstraps or more are shown. The scale bar shows the number of amino acid differences per site. Subclades containing MoHOXs are shaded, in which MoHOXs and characterized genes from other fungi are shown in bold in red or black, respectively. Green and purple boxes in the domain architecture represent the homeodomains IPR001356 and IPR003120, respectively, and orange boxes are C_2_H_2_ zinc finger motifs (IPR007087). Abbreviations for fungal species followed by their GenBank accession numbers are as follows: Mo, *Magnaporthe oryzae*; Sc, *Saccharomyces cerevisiae*; Cg, *Candida glabrata*; Ca, *Candida albicans*; Fg, *Fusarium graminearum*; Tr, *Trichoderma reesei*; Nc, *Neurospora crassa*; Kl, *Kluyveromyces lactis*; CnB, *Cryptococcus neoformans* serotype D B3501-A; Lb, *Laccaria bicolor*; Pc, *Phanerochaete chrysosporium*; An, *Aspergillus nidulans*; Pm, *Penicillium marneffeip*; Pa, *Podospora anserine*; Um, *Ustilago maydis*; Pr, *Phytophthora ramorum*; Ps, *Phytophthora sojae*; CnJ, *Cryptococcus neoformans* serotype D JEC 21; Ec, *Encephalitozoon cuniculi*; Yl, *Yarrowia lipolytica*; Sn, *Stagonospora nodorum*; Fo, *Fusarium oxysporum*; Fs, *Fusarium solani*; Cc, *Coprinus cinereus*.

Members of the homeobox TF family possess a conserved DNA-binding motif called the homeodomain [40]. Using InterPro terms (IPR001356 and IPR003120) for homeodomains, 216 homeobox TFs were retrieved from 22 eukaryotic microbe genomes, including eight (*MoHOX1* to *MoHOX8*) in *M. oryzae* (Table S1). The *MoHOX* genes separated into eight distinct clades in the phylogenetic tree (Figure 1). This suggests that expansion of the DNA-binding TF family may be linked to functional divergence, as hypothesized previously [41]. The *MoHOX*-clades, except the *MoHOX8*-clade, which contains a divergent form of the homeodomain, embrace homeobox TFs belonging only to the subphylum Pezizomycotina, but not to the subphylum Saccharomycotina or phylum Basidiomycota, suggesting that homeobox TFs have evolved in a lineage-specific manner [41],[42].

### Generation of *MoHOX* deletion mutants and observation of their phenotypes

In order to characterize the roles of homeobox genes in *M. oryzae* development and pathogenicity, constructs for the targeted disruption of the *MoHOX* genes were generated using a split-marker deletion method or double-joint PCR method (Figure S1). Eight deletion mutants were generated in which all of the *MoHOX* genes were replaced with a hygromycin resistance cassette, as confirmed by DNA blot and RT-PCR analyses using a gene-specific probe and sets of PCR primers (Figure S1, Table S2). The strains of the wild-type and transformants generated in this study are presented in Table 1.

**Table 1:** Various fungal strains used in this study.

Strains	Genotypes	Reference
KJ201	Wild-type	This study
70-15	Wild-type	"
Guy11	Wild-type	"
*ΔMohox1*	*MoHOX1* deletion mutant of KJ201	"
*MoHOX1e*	Ectopic transformant with *MoHOX1* deletion construct	"
*ΔMohox2*	*MoHOX2* deletion mutant of KJ201	"
*MoHOX2e*	Ectopic transformant with *MoHOX2* deletion construct	"
*Mohox2c*	Complemented transformant of *ΔMohox2* mutant	"
*ΔMohox3*	*MoHOX3*deletion mutant of KJ201	"
*MoHOX3e*	Ectopic transformant with *MoHOX3* deletion construct	"
*ΔMohox4*	*MoHOX4*deletion mutant of KJ201	"
*MoHOX4e*	Ectopic transformant with *MoHOX4* deletion construct	"
*ΔMohox5*	*MoHOX5*deletion mutant of KJ201	"
*MoHOX5e*	Ectopic transformant with *MoHOX5* deletion construct	"
*ΔMohox6*	*MoHOX6* deletion mutant of KJ201	"
*MoHOX6e*	Ectopic transformant with *MoHOX6* deletion construct	"
*ΔMohox7*	*MoHOX7* deletion mutant of KJ201	"
*MoHOX7e*	Ectopic transformant with *MoHOX7* deletion construct	"
*Mohox7c*	Complemented transformant of *ΔMohox7* mutant	"
*ΔMohox8*	*MoHOX8* deletion mutant of KJ201	"
*MoHOX8e*	Ectopic transformant with *MoHOX8* deletion construct	"
*ΔMocrz1*	*MoCRZ1* deletion mutant of KJ201	[10]
*Δmck1*	*MCK1* deletion mutant of KJ201	[7]
*ΔMoplc1*	*MoPLC1* deletion mutant of 70-15	[17]
*Δcpka*	*CPKA* deletion mutant of 70-15	[9]
*Δmac1*	*MAC1* deletion mutant of 70-15	[12]
*Δpmk1*	*PMK1* deletion mutant of Guy11	[6]

The effects of the deletion of *MoHOX* genes on *M. oryzae* development and pathogenicity are summarized in Table 2. In brief, deletion mutants of each *MoHOX* gene exhibited unique phenotypes in fungal development and pathogenicity, such as mycelial growth, conidial morphology, conidiation, and appressorium formation. The *ΔMohox1* mutant showed a significant reduction in vegetative growth, but increase in melanin pigmentation (Figure S2). The *ΔMohox6* mutant also exhibited a significant reduction in vegetative growth, whereas other phenotypes in the *ΔMohox6* mutant were indistinguishable from those of the wild-type. Conidiation (asexual reproduction) was completely abrogated in the *ΔMohox2* mutant, but this defect was recovered when the wild-type copy of the *MoHOX2* gene was transformed into the mutant. The *ΔMohox4* mutant produced shorter and smaller conidia in both length and width compared to those of the wild-type (Figure S3). The *ΔMohox7* mutant was unable to form appressoria on hydrophobic surfaces, while its other phenotypes were similar to those of the wild-type. However, the *ΔMohox8* mutant formed appressoria, but was nonpathogenic due to a defect in penetration. The *mst12* mutant, carrying a partial deletion in the *MST12* gene, shows the same phenotypes as the *ΔMohox8* mutant, confirming that they are the same gene [30]. No distinguishable phenotypes were observed in *ΔMohox3* and *ΔMohox5*, as compared to the wild-type. Based on our phenotypic observations of *MoHOX* deletion mutants, the mutants *ΔMohox2* and *ΔMohox7* were chosen for detailed studies as their phenotypes are associated with important developmental stages in the *M. oryzae* disease cycle.

**Table 2:** Characterization of transformants including deletion mutants for *MoHOX* genes in *M. oryzae* development and pathogenicity.

			Conidium size^c^				
Strain	Growth (mm)^a^	Conidiation (10^5^/ml)^b^	Length (µm)	Width (µm)	Germination (%)^d^	Appressorium formation (%)^e^	Pathogenicity (Disease index)^f^
KJ201	66.4±0.5^Dg^	118.3±11.9^BC^	32.1±2.7^D^	9.8±1.2^D^	96.2±1.4^AB^	98.5±1.1^C^	7.5±1.8^B^
*ΔMohox1*	29.9±0.6^A^	99.0±3.0^BC^	31.6±3.8^D^	9.3±0.8^C^	96.5±0.7^AB^	99.4±0.5^C^	7.0±0^B^
*MoHOX1e*	59.0±2.5^CD^	108.7±4.9^BC^	31.4±3.0^D^	9.2±0.9^C^	97.9±0.7^AB^	97.7±0.6^C^	6.3±1.2^B^
*ΔMohox2*	66.1±0.9^CD^	0^A^	ND^h^	ND	ND	ND	ND
*MoHOX2e*	65.4±2.1^CD^	92.7±5.5^B^	30.9±3.0^BC^	9.7±1.3^D^	94.5±1.4^A^	99.4±1.1^C^	7.7±1.2^B^
*Mohox2c*	63.2±0.8^CD^	104±10.4^BC^	31.7±2.3^D^	8.9±0.8^AB^	98.5±0.4^B^	98.2±1.0^C^	8.0±0.8^B^
*ΔMohox3*	58.8±5.7^CD^	100±11.1^BC^	30.1±3.2^BC^	9.1±0.9^BC^	95.3±1.7^AB^	98.0±0.5^C^	7.0±2.2^B^
*MoHOX3e*	64.3±4.2^CD^	93.0±9.5^B^	30.8±3.2^BC^	9.3±1.1^C^	97.4±0.6^AB^	98.3±1.2^C^	8.0±2.0^B^
*ΔMohox4*	64.8±1.0^CD^	112.3±4.6^BC^	27.3±3.0^A^	8.7±1.0^A^	97.3±0.7^AB^	98.5±1.3^C^	6.0±1.2^B^
*MoHOX4e*	63.4±5.0^CD^	104.7±9.7^BC^	31.1±3.3^CD^	9.8±1.1^D^	97.3±0.8^AB^	98.2±0.9^C^	8.0±0.8^B^
*ΔMohox5*	61.6±2.9^CD^	102.0±11.8^BC^	31.4±3.2^D^	9.7±0.8^D^	98.8±0.6^B^	99.4±1.0^C^	6.0±1.7^B^
*MoHOX5e*	63.3±6.2^CD^	118.0±6.6^BC^	31.9±3.2^D^	9.2±0.9^C^	97.9±0.7^AB^	98.8±0.8^C^	6.6±1.7^B^
*ΔMohox6*	57.8±3.8^B^	125.0±8.2^BC^	31.3±3.3^CD^	9.3±1.0^C^	98.1±1.1^B^	98.8±1.1^C^	5.0±0^B^
*MoHOX6e*	64.4±2.3^CD^	116.3±15.5^BC^	30.8±2.6^BC^	9.7±1.0^D^	98.2±1.8^B^	99.2±0.8^C^	7.5±1.0^B^
*ΔMohox7*	65.4±0.5^CD^	99.3±18.2^BC^	32.2±2.8^D^	9.0±0.8^AB^	96.9±2.3^AB^	0.3±0.5^A^	0^A^
*MoHOX7e*	58.0±3.6^BC^	119.3±15.5^BC^	31.6±2.5^D^	9.8±1.1^D^	97.8±1.5^AB^	99.0±1.0^C^	6.3±1.2^B^
*Mohox7c*	65.3±1.0^CD^	99.5±10.4^BC^	31.3±3.0^CD^	8.9±0.9^AB^	98.8±0.6^B^	9.8±0.8^B^	7.4±2.2^B^
*ΔMohox8*	62.3±2.8^CD^	106.7±19.6^BC^	29.4±2.9^B^	8.9±0.9^AB^	98.7±0.4^B^	97.3±0.3^C^	0^A^
*MoHOX8e*	62.6±1.8^CD^	130.0±6.6^C^	29.9±3.3^B^	8.9±1.0^AB^	97.1±0.2^AB^	99.4±0.5^C^	8.0±2.0^B^

a Vegetative growth was measured at 10 days post-inoculation on complete agar medium. Data were presented as means±SD from three independent experiments.

b Conidiation was measured by counting the number of conidia collected with 5 ml of sterilized distilled water from 10-days-old oatmeal agar plates.

c Sizes of conidia were determined from at least two experiments with over 100 conidia each.

d Percentage of conidial germination on hydrophobic surfaces was measured under a light microscope using conidia harvested from 6-days-old V8 juice agar plates.

e Percentage of appressorium formation on hydrophobic surfaces was measured using conidia harvested from 6-day-old V8 juice agar plates.

f Scores of disease severity was measured 7 days after inoculation, as previously described [63].

g Tukey's test was used to determine significance at the 95% probability level. The same letters in a column showed no significant difference.

h ND = not determined.

### *MoHOX2* is essential for conidiogenesis

**Figure 2:**
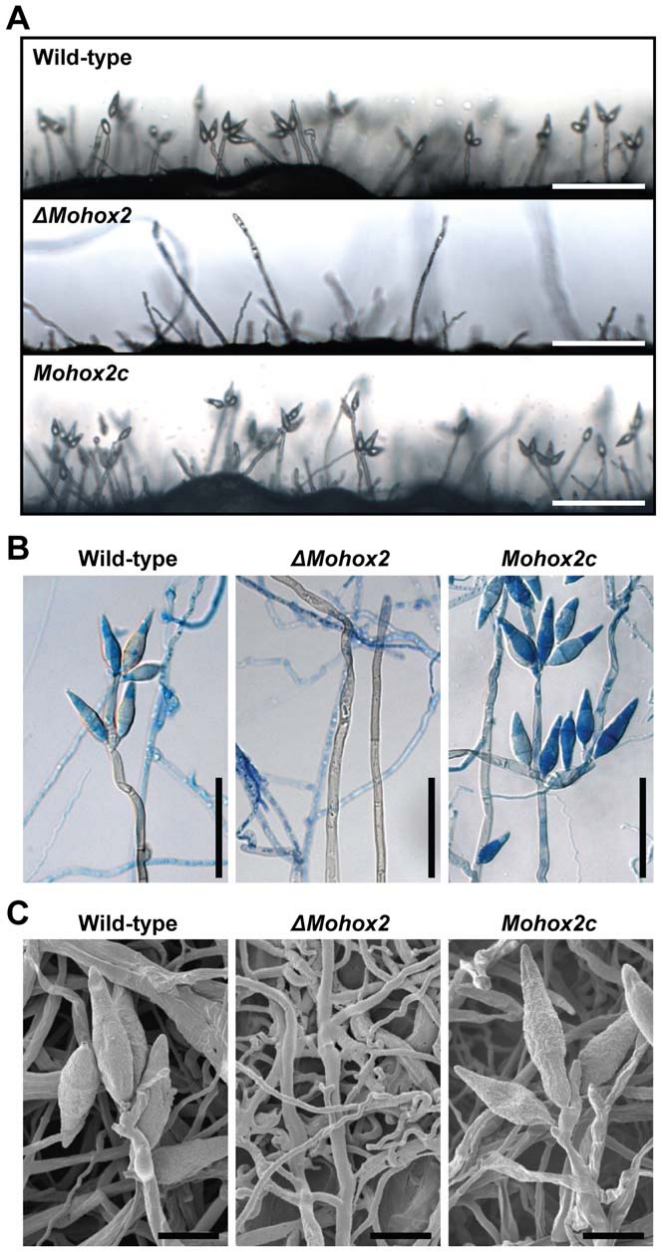
Effect of *MoHOX2* deletion on conidiogenesis. (A) Development of conidia on conidiophores. Light microscopic observation was performed on strains grown on V8 medium for 7 days. Bars = 100 µm. (B) Aerial structures stained with lactophenol aniline blue. Conidia and aerial hyphae stained blue, and conidiophores stained gray. Bars = 50 µm. (C) Scanned electron microscopic observation of aerial structures. Bars = 10 µm.

Quantitative measurement of conidia reconfirmed that conidial production was completely abolished in the *ΔMohox2* mutant on V8 juice or oatmeal agar media. However, this defect in conidiation was fully recovered in the complemented transformant *Mohox2c*, to a similar extent as in the wild-type (Table 2). The other phenotypes in the *ΔMohox2* mutant were quite similar to those in the wild-type and complemented transformant *Mohox2c* (Table 2, Figure S4), indicating that MoHOX2 is specifically involved in conidiation. Microscopic observation was performed to carefully define the effect of the *MoHOX2* deletion on conidial formation (Figure 2). The wild-type and *Mohox2c* developed pear-shaped conidia on a conidiophore in a sympodial pattern 18 h after incubation (Figure 2A and 2C). However, no conidia formed in *ΔMohox2* after prolonged incubation under conidial induction conditions, even though conidiophore development appeared to be normal in the mutant. In order to determine if *MoHOX2* is also involved in conidiophore development, we stained patches of aerial mycelia with lactophenol aniline blue to distinguish conidiophores from other aerial hyphae [27]. Microscopic examination revealed that conidiophores developed in the *ΔMohox2* mutant, as in the wild-type and *Mohox2c* (Figure 2B). These results indicate that *MoHOX2* is a specific regulator that is essential for conidial development.

### *MoHOX2* is required for conidiogenesis but not for appressorium-related pathogenic development

**Figure 3:**
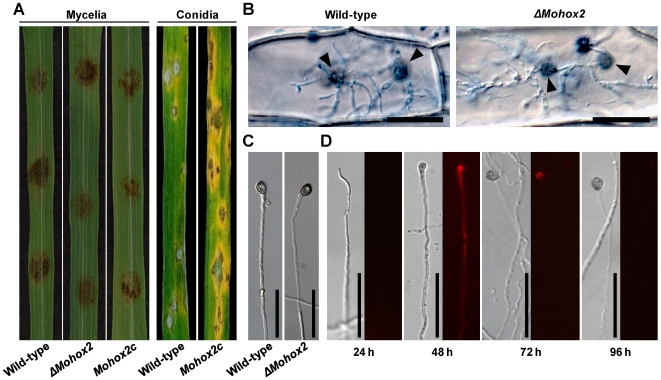
Effect of *MoHOX2* deletion on appressorium development and pathogenicity. (A) Assay for pathogenicity. Intact rice leaves were inoculated either by placement of agar plugs containing mycelia (6 mm in diameter) or by spraying conidial suspension (10^5^ conidia/ml) of the indicated strains. (B) Assay for hypha-mediated penetration. Onion epidermis was inoculated with a patch of mycelia of the wild-type or *ΔMohox2*. Black arrowheads indicate sites of penetration *via* hyphal appressoria. Bars = 50 µm. (C) Appressorium development at hyphal tips. Mycelial blocks were placed on hydrophobic surfaces, and incubated for 72 h at 25°C. Bars = 50 µm. (D) Microscopic observation of temporal and spatial occurrence of lipid droplets during hypha-driven appressorium development. Mycelial blocks of the wild-type were incubated on hydrophobic surfaces, and stained with Nile red to visualize the formation of lipid droplets. Note that lipid droplets were not initially detected in hypha until 24 h, but became abundant in hypha with developing apprssorium 48 h after inoculation. Bars = 50 µm.

To evaluate the role of *MoHOX2* during *M. oryzae* disease development, a pathogenicity assay was performed by inoculating susceptible rice leaves with mycelial agar plugs, rather than a conidial suspension, because *ΔMohox2* is unable to produce conidia. The inoculation with *ΔMohox2* mycelial blocks caused blast lesions similar to the wild-type (Figure 3A). Given that successful lesion development requires the development of appressoria on germ tubes of conidia for penetration into plant cells, this result led us to examine diseased tissue using microscopy. As expected, many appressorium-like structures were observed on the surfaces of rice leaves inoculated with either *ΔMohox2* or wild-type mycelial agar plugs (data not shown). To investigate whether these appressorium-like structures contribute to disease development, we carried out a penetration assay, in which a mycelial agar plug (6 mm in diameter) was placed on onion epidermal cells. Both the wild-type and *ΔMohox2* developed appressoria specifically at tips of hyphae on onion cells and invasive hyphal growth was subsequently observed underneath the appressorium inside the cell (Figure 3B). Appressoria that formed on hyphal tips of the wild-type and *ΔMohox2* were indistinguishable in shape, size, and melanization. In order to evaluate whether a hydrophobic surface is conductive to appressorial formation by hyphae, an agar plug containing actively growing hyphae was placed on either the hydrophobic or hydrophilic surface of Gelbond film. Unexpectedly, both surfaces induced appressorial formation at hyphal tips of the wild-type and *ΔMohox2* (Figure 3C). As seen in pathogenicity assays with *ΔMohox2* mycelia, conidia from *Mohox2c* caused typical necrotic lesions on foliar parts of the host plant, similar to those caused by the wild-type (Figure 3A).

During appressorium-mediated penetration, lipid droplets abundant in conidium move into the incipient appressorium and degrade at the onset of turgor generation [43]. To understand the functional role of hypha-driven appressoria, we examined the temporal and spatial occurrence of lipid droplets using Nile red staining and epifluorescence microscopy. Unlike in conidia, lipid droplets were not initially detected in hyphae of the wild-type or *ΔMohox2* on appressorium-inductive surfaces until 24 h (Figure 3D). However, lipid droplets became abundant in hyphae, and translocated into nascent appressoria 48 h after inoculation. The process was completed by 72 h. Considering that translocation of lipid droplets into nascent appressoria on conidial germ tubes occurs within 4 h [43], such a delayed process seems to be associated with the *de novo* synthesis of lipid droplets and hypha-driven appressorium development. Taken together, these results indicate that *MoHOX2* is essential for conidiogenesis, but dispensable for appressorium formation and pathogenicity. Also, *M. oryzae* can form hypha-driven appressoria that can cause foliar disease.

### Transcriptional expression of *MoHOX2* and conidiogenesis-related genes

**Figure 4:**
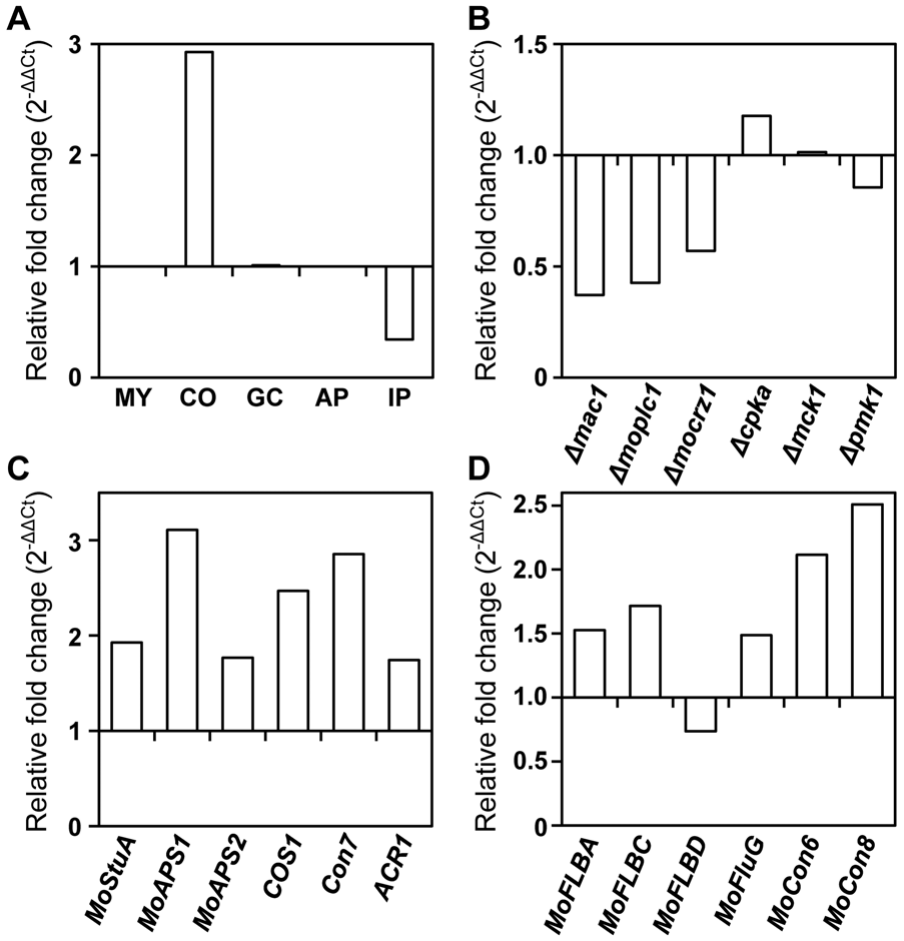
Transcriptional expression patterns of *MoHOX2* and conidiogenesis-related genes. (A) Expression of *MoHOX2* during *M. oryzae* development. The tissues examined were submerged mycelia (MY), purified conidia (CO), germinated conidia (GC), appressoria (AP), and infected plants (IP). Measurements of *MoHOX2* transcripts obtained by quantitative RT-PCR analysis were normalized to *β-tubulin* and expressed as relative values, with 1 corresponding to the MY. See Materials and Methods for details. (B) Expression of *MoHOX2* in various deletion mutants. These include *Δmac1*, *Δmoplc1*, *Δmocrz1*, *Δcpka1*, *Δmck1*, and *Δpmk1*, and their genotypes were described in Table 1. The abundance of *MoHOX2* transcripts in a mutant is expressed relative to a value of 1 in the wild-type KJ201. RNAs of the mutants were extracted from mycelia grown in CM liquid medium for 4 days. (C) Expression of conidiogenesis-related *M. oryzae* genes. The abundance of conidiogenesis-related transcripts in *ΔMohox2* is expressed as a relative value, with 1 corresponding to *MoHOX2* transcripts in the wild-type. (D) Expression of *M. oryzae* genes homologous to known conidiogenesis-related genes in other fungi. The abundance of conidiogenesis-related transcripts in *ΔMohox2* is expressed as a relative value, with 1 corresponding to the level of *MoHOX2* transcripts in the wild-type.

Quantitative real-time RT-PCR (qRT-PCR) was used to examine the expression pattern of *MoHOX2* under various conditions. The *MoHOX2* gene was found to be constitutively expressed during development (Figure 4A). *MoHOX2* transcript levels were dramatically higher during conidiation but lower during invasive growth, as compared to other stages. This pattern of *MoHOX2* expression appears to be correlated with a functional role for MoHOX2 in conidiation.

To evaluate the impact of upstream signaling pathways on *MoHOX2* expression, we measured the expression levels of the *MoHOX2* gene in mutant backgrounds related to signal transduction (Figure 4B, Table 1). Significantly reduced *MoHOX2* expression (greater than two-fold) was found in the adenylate cyclase mutant *Δmac1* and the phospholipase C mutant *ΔMoplc1*. The expression of the *MoHOX2* gene changed slightly, but not significantly, less than two-fold in other mutants, including the mutants *Δmocrz1* for a calcineurin-responsive TF, *ΔcpkA* for a cAMP-dependent protein kinase catalytic subunit, *Δmck1* for a MAPKKK, and *Δpmk1* for a MAPK. These results suggest that the expression of *MoHOX2* is co-regulated by cAMP and Ca^2+^-dependent pathways.

Since MoHOX2 is a putative homeobox TF, specifically required for an earlier stage of conidiation, it is reasonable to speculate that MoHOX2 acts as a TF that modulates the expression of other conidiation-related genes. To determine the impact of *MoHOX2* deletion on the expression of conidiation-related *M. oryzae* genes and other *M. oryzae* genes that are orthologs to conidiation-related genes in other fungi (Table 3), their expression levels were measured in the *ΔMohox2* mutant background. The expression of the genes *MoAPS1*, *COS1*, and *Con7* was significantly upregulated (Figure 4C), as were the *M. oryzae* genes *MoCon6* and *MoCon8*, orthologs to *N. crassa Con-6* and *Con-8* (Figure 4D). These results indicate that genes tested are not direct targets of MoHOX2. However, the altered levels of gene expression in *ΔMohox2* suggest that *MoHOX2* functions as a key TF of downstream gene expression leading to conidiogenesis.

**Table 3:** *Magnaporthe oryzae* genes used in quantitative real-time PCR.

Gene	Locus number	Description, mutant phenotype	Reference
*COS1*	MGG_03394.6	C_2_H_2_ zinc finger transcription factor (TF), conidiophores stalk-less	[27]
*CON7*	MGG_05287.6	C_2_H_2_ zinc finger TF, abnormal conidia	[24]
*ACR1*	MGG_09847.6	Hypothetical protein with a glutamine-rich domain, acropetal conidia	[23]
*MoSTUA*	MGG_00692.6	APSES TF, severe reduction in conidial production	Unpublished data
*MoAPS1*	MGG_09869.6	APSES TF, reduction in conidial production	"
*MoAPS2*	MGG_08463.6	APSES TF, reduction in conidial production	"
*MoFLBA*	MGG_14517.5	Putative regulator of G protein signalling, ortholog to *flbA* in *Aspergiluus nidulans*	[69]
*MoFLBC*	MGG_04699.6	C_2_H_2_ zinc finger TF, ortholog to *flbC* in *A. nidulans*	[69]
*MoFLBD*	MGG_06898.6	MYB TF, ortholog to *flbD* in *A. nidulans*	[69]
*MoFLUG*	MGG_02538.6	Putative glutamine synthetase, ortholog to *fluG* in *A. nidulans*	[70]
*MoCON6*	MGG_02246.6	Hypothetical protein, ortholog to *con-6* in *Neurospora crassa*	[71]
*MoCON8*	MGG_00513.6	Hypothetical protein, ortholog to *con-8* in *N. crassa*	[71]

### *MoHOX7* is essential for appressorium formation

**Figure 5:**
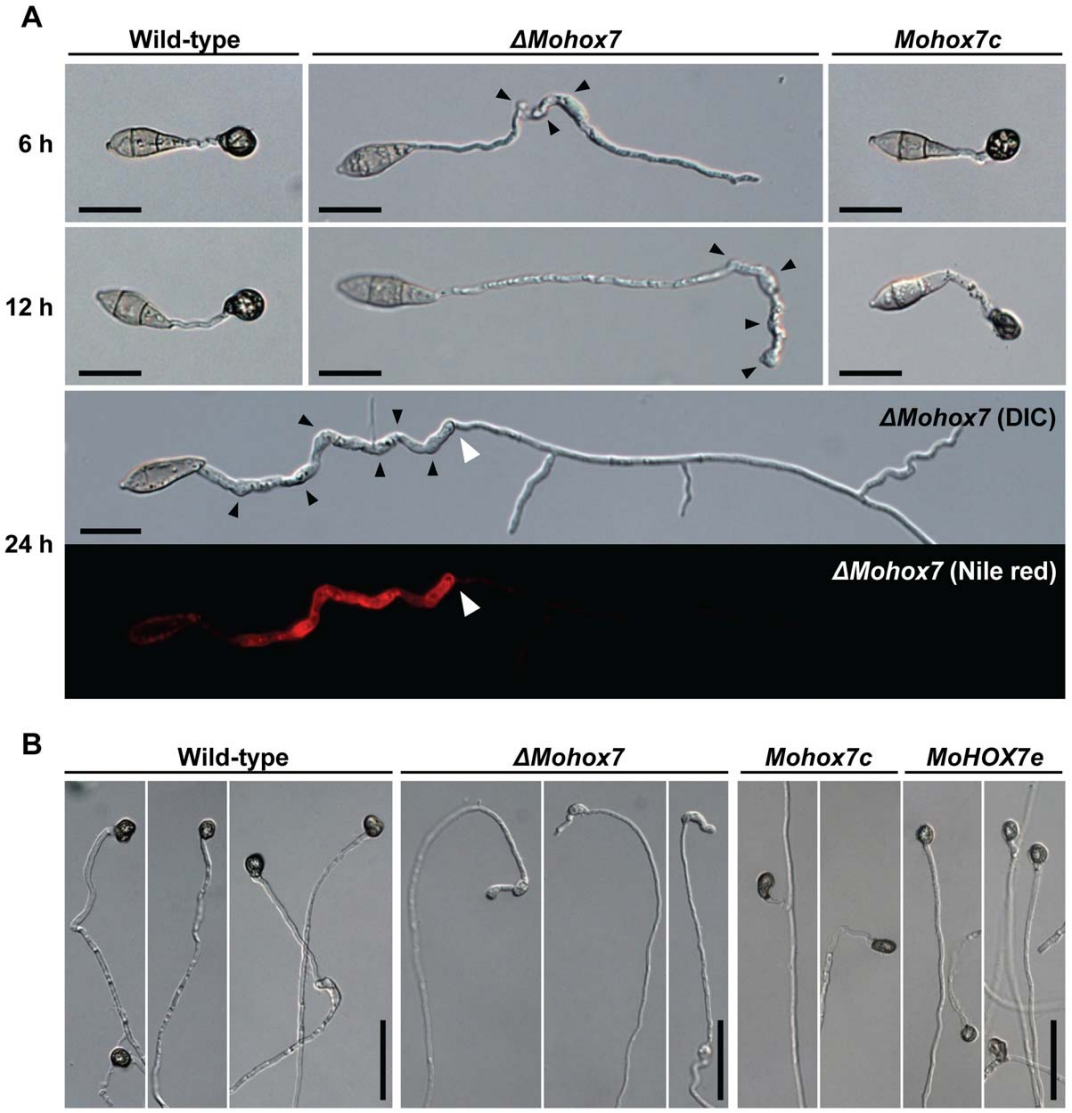
Effect of *MoHOX7* deletion on appressorium formation. (A) Microscopic observation of appressorium development on germ tubes. Appressorium formation was examined at 6, 12, and 24 h after incubation of the conidial suspension on hydrophobic cover slips. Black arrowheads in *ΔMohox7* indicate the occurrence of swellings and hooks in a germ tube. Upon prolonged incubation of *ΔMohox7* in the bottom panel, its germ tube initiated vegetative growth from a site indicated with a white arrowhead. The disappearance of lipid droplets after vegetative growth was revealed using the fluorescent dye Nile red. Bars = 20 µm. (B) Appressorium development on hyphae. Appressorium formation was induced by placing mycelia blocks on a hydrophobic plastic surface for 72 h. Bars = 50 µm.

The deletion of *MoHOX7* completely abolished the ability to form appressoria while other phenotypes were not affected (Table 2). Microscopic examination revealed that wild-type conidia formed appressoria on the tip ends of germ tubes 6 h after incubation on a hydrophobic surface (Figure 5A). In contrast, the *ΔMohox7* mutant failed to develop appressoria; instead, the germ tubes in *ΔMohox7* elongated abnormally and appeared to have several rounds of swellings and hooking until 12 h. During prolonged incubation (24 h) of the *ΔMohox7* mutant, its germ tubes grew like vegetative hyphae, with branches rather than repeating recognition steps (Figure 5A). Nile red staining of germ tubes of the *ΔMohox7* mutant revealed a large accumulation of lipid droplets until swelling and hooking occurred, before switching to vegetative hyphal growth (Figure 5A). These defects in the *ΔMohox7* mutant were repaired in the complemented transformant *Mohox7c*. This suggests that *MoHOX7* plays a critical role in appressorium development rather than the recognition of cues that induce appressorium formation.

Next, we tested whether *MoHOX7* is also required to form appressoria at hyphal tips. Not surprisingly, the *ΔMohox7* mutant did not form an appressorium on hydrophobic and hydrophilic surfaces, although non-melanized swellings and hooking were found on tips of hyphae (Figure 5B), as observed with conidial germ tubes in Figure 5A. In contrast, the wild-type, *Mohox7c*, *and MoHOX7e* formed appressoria at the tips of mycelia (Figure 5A). This supports the idea that *MoHOX7* is essential for the development of appressoria, both from hyphae and germ tubes.

### *MoHOX7* is not necessary for invasive growth

**Figure 6:**
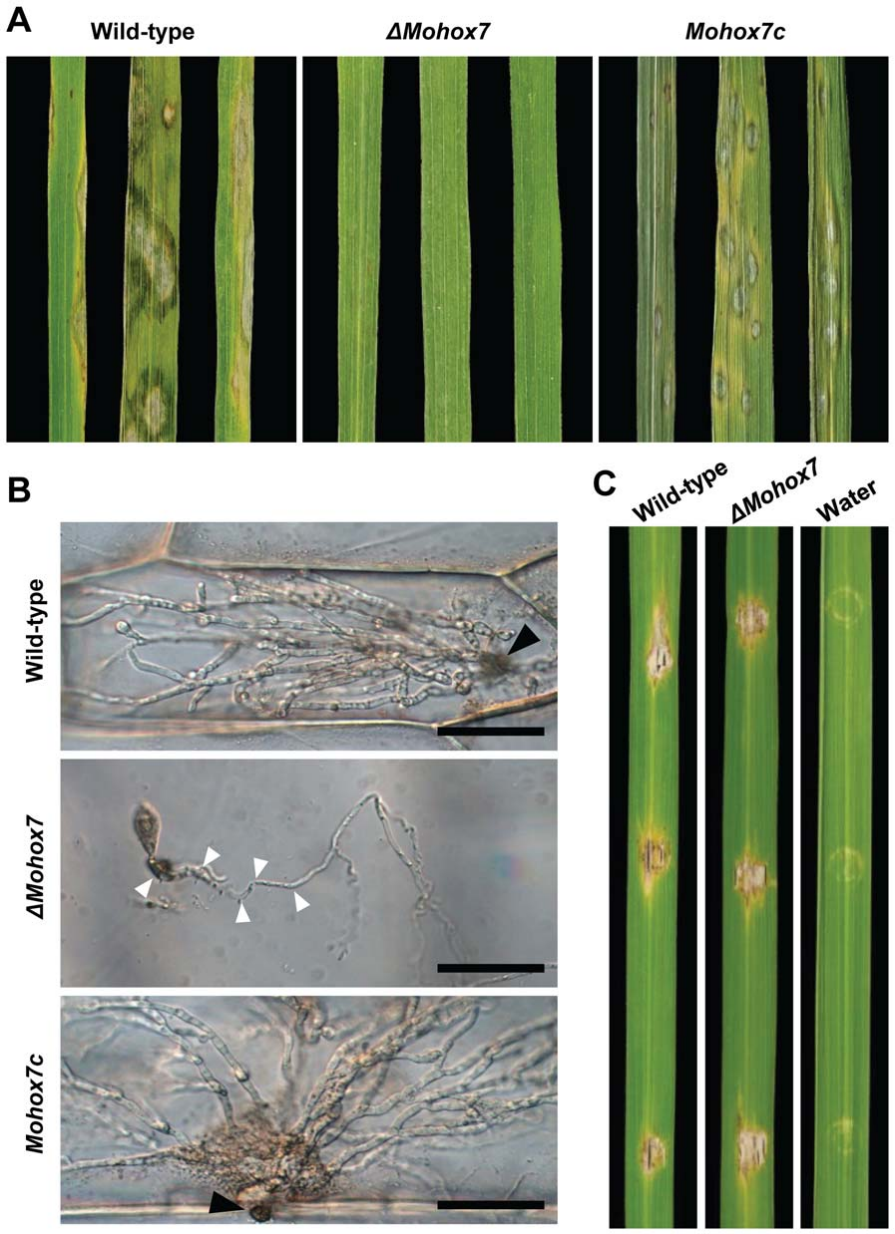
Effect of *MoHOX7* deletion on pathogenicity. (A) Assay for pathogenicity. Conidial suspensions (10^5^ conidia/ml) of the indicated strains were sprayed on 3-week-old intact rice leaves. Photographs were taken 7 days after inoculation. (B) Assay for penetration. 40 µl of conidial suspension (3×10^4^/ml) of the strains were inoculated on onion epidermal cells for 48 h. (C) Infiltration assay for growth inside plant cells. Conidial suspensions (5×10^4^ conidia/ml) of the indicated strains were infiltrated into rice leaves using a syringe. Water served as control. Photographs were taken 7 days after inoculation.

Infection assays on rice were performed to test whether the *ΔMohox7* mutant can cause disease on host tissues. Conidial suspensions were sprayed onto 3-week-old rice seedlings. The wild-type caused blast lesions on the plant, but the *ΔMohox7* mutant produced no lesions in plant cells (Figure 6A). These defects in the *ΔMohox7* mutant were fully restored to the wild-type levels in *Mohox7c*. To test the role of *MoHOX7* in penetration, onion epidermis and rice leaf sheath cells were inoculated with a conidial suspension. The wild-type penetrated into epidermal cells and grew invasively (Figure 6B). In contrast, the *ΔMohox7* mutant was unable to penetrate into plant surfaces. Germ tubes appeared to have rounds of swellings and hooking before vegetative growth began on plant surfaces (Figure 6B). To determine whether the *ΔMohox7* mutant can perform invasive growth in plant cells, a conidial suspension was infiltrated into rice leaves by injection using a syringe. Both the wild-type and *ΔMohox7* developed blast lesions, indicating that the mutant still had the ability to grow inside plant cells (Figure 6C). These results suggest that the functioning of *MoHOX7* is not required for further growth inside the host, and is strictly limited to the stage of appressorial development.

### The expression of *MoHOX7* is affected by cAMP and Ca^2+^-dependent signaling pathways

**Figure 7:**
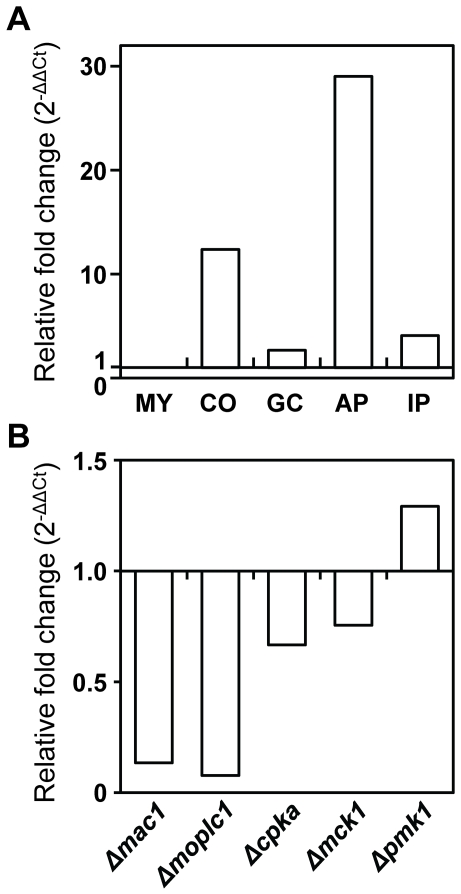
Expression patterns of *MoHOX7*. (A) Expression of *MoHOX7* during *M. oryzae* development. Abbreviations for the tissues used are shown in Figure 4A. The levels of *MoHOX7* transcripts in a quantitative RT–PCR analysis were normalized to *β-tubulin*, and are expressed as relative values with 1 corresponding to the MY. (B) Expression of *MoHOX7* in various deletion mutants. These include *Δmac1*, *Δmoplc1*, *Δcpka1*, *Δmck1*, and *Δpmk1*, and their genotypes were described in Table 1. The abundance of *MoHOX7* transcripts in each mutant is expressed as a value relative to 1 in the wild-type KJ201.

qRT-PCR was performed to determine the transcription level of *MoHOX7* during developmental stages in *M. oryzae*. The transcription of *MoHOX7* was dramatically upregulated during appressorium formation (>29-fold), conidiation (>12-fold), and in invasive growth *in planta* (>four-fold) at 78 h after inoculation, compared to its expressions during mycelial growth (Figure 7A). The highest expression of *MoHOX7* during appressorium development was consistent with its role in appressorium-mediated disease development, as shown in Figure 5.

To investigate whether signaling pathways associated with appressorium-mediated penetration affect the expression of the *MoHOX7* gene, the levels of *MoHOX7* transcripts were measured in several mutants related to signal transduction (Table 1, Figure 7B). Much lower levels of *MoHOX7* transcripts were observed in *ΔMoplc1* and *Δmac1* mutants, whereas the expression of the *MoHOX7* gene was not significantly affected in the mutants *Δcpka*, *Δmck1*, or *Δpmk1*. This suggests that the expression of *MoHOX7* is regulated by Ca^2+^ and cAMP-dependent signaling pathways (Figure 7B). To test if such signaling molecules can restore appressorium formation in the *ΔMohox7* mutant, a conidial suspension of *ΔMohox7* on hydrophilic and hydrophobic surfaces was treated with the chemicals 1,16-hexadecanediol (HDD), cAMP, CaCl_2_, and 1,2-dioctanoyl-sn-glycerol (DOG), a diacylglycerol analogue. None of these molecules complemented the defects in appressorium formation in the *ΔMohox7* mutant on both the hydrophilic and hydrophobic surfaces, while these molecules increased appressorium formation in the wild-type on the hydrophilic surface (Table S3). This result suggests that *MoHOX7* is a key downstream regulator of appressorium development.

## Discussion

Conidiogenesis and appressorium development are key steps in the colonization of host plants by many fungal pathogens. These processes are controlled by a precise developmental program in response to stimuli from the host and environment. Organisms have evolved regulatory networks to ensure the correct timing and spatial pattern of the developmental events. Transcription factors (TFs) play important roles in fungal development and pathogenicity as regulators in biological networks. The functional analysis of TFs provides new insights into a controlling network that governs fungal development and pathogenicity.

In an effort to understand developmental biology in *M. oryzae*, we identified eight homeobox TFs (*MoHOX1 to MoHOX8*) as candidates for development-controlling genes since they are well-known regulators of development and differentiation in other organisms [29],[35],[36]. *MoHOX2* proved essential for conidiogenesis. The disruption of *MoHOX2* completely abolished the ability of *M. oryzae* to produce conidia, even though conidiophore development was normal. The deletion of this gene did not affect any other developmental stages, such as hyphal growth, appressorium formation, and penetration, except for a subtle difference. Our study suggests that MoHOX2 is a stage-specific regulator of an earlier step of conidiogenesis. This phenotypic feature in the mutant is interesting and unique because the deletion of any of the other genes related to conidiogenesis caused pleiotropic defects in *M. oryzae*. The *Δcon7* mutant, affected in a zinc finger TF, develops morphologically abnormal conidia and never forms an appressorium [24]. A very recent study reported the interesting finding that *COS1*, which encodes another zinc finger TF, is a determinant of conidiophore formation and melanin pigmentation [27]. The authors also observed that the *Δcos1* mutant, unlike the wild-type, developed appressorium-like structures on the host surface and disease symptoms in a mycelial inoculation test, and speculated that COS1 may have a role in an unknown mechanism involved in mycelia-mediated infection. However, we believe that the wild-type can form appressoria at hyphal tips as a strategy for host infection, although there may be strain-dependent differences. Such hypha-driven appressoria in the *ΔMohox2* and wild-type mediated penetration into host cells caused typical symptoms of rice blast, indicating a functional similarity to conidial germ tube-driven appressoria in *M. oryzae* disease development. Hyphal appressorium-mediated penetration by *M. oryzae* may not predominantly occur in nature due to limitations in the spatial distribution of hyphae and on hyphal longevity. However, members of non-sporulating fungi develop sclerotia as survival and inoculum structures, in which hyphae become interwoven, aggregated, melanized, and dehydrated [44],[45]. These fungal pathogens penetrate host epidermal cells by means of infection cushions, aggregated forms of branched hyphae, after their perception of host factors [46]. The *ΔMohox7* mutant, defective in appressorium formation on conidial germ tubes, was also unable to form appressoria at hyphal tips, and the defect was not recovered with the addition of exogenous cAMP. This indicates that *MoHOX7* regulates appressorium development in both hyphae and conidial germ tubes. The role of *MoHOX7* in appressorium development has been predicted by the study of a large deletion mutant, *Δpth12* (NCBI accession number DQ060925), which was generated by restriction enzyme-mediated integration (REMI), but no detailed characterization of *Δpth12* has been performed. Although entry into the host is possible through natural surface openings such as stomata, this is not believed to be the predominant mode for spread of infection; the *ΔMohox7* mutant consistently failed to colonize unwounded host leaves. Independent of appressorium-mediated infection, *M. oryzae* has evolved other strategies to cause disease on hosts. During root infection, hyphae swellings resembling the hyphopodia of root-infecting fungi are associated with root invasion, leading to systemic disease development similar to typical foliar disease [47]. This evidence supports the idea that *M. oryzae* has evolved hypha-mediated infection structures to gain entry into their hosts.

A variety of signals induce appressorium formation, including surface hardness, hydrophobicity, adhesion quality, and host molecules [48]–[51]. The hyphae of the *ΔMohox2* mutant and wild-type developed appressoria upon sensing hydrophobic surfaces, consistent with a previous observation [52]. In addition, the hyphae were also able to form appressoria on hydrophilic surfaces, unlike conidial germ tubes. This suggests that there is an unknown pathway that regulates the development of the hypha-driven appressoria after sensing environmental cues such as the surface hardness. The strong adhesion of conidial germ tubes to substrates is needed for appressorial development, and we also observed that tight adhesion of hyphae is a prerequisite to formation of appressoria at the tips. Adhesion, therefore, appears to be a critical step prior to the initiation of infection, not only for the prevention of dislodgement, but also for subsequent correct development. After perceiving inductive signals, germ tubes of conidia exhibit hooking and swelling related to appressorium formation. Thus, the continued hooking and swelling in the *ΔMohox7* mutant indicates that MoHOX7 is not involved in signal recognition. In contrast, the deletion of *MagB*, which encodes a Gα subunit involved in sensing a surface cue, causes the formation of long and straight germ tubes [15].

The biological processes that mediate a functional appressorium from a conidial germ tube are very complicated, based on molecular and cytological evidence such as autophagy [53], changes in metabolism [43], cell cycle control [53], and the generation of turgor pressure [5]. A body of evidence has demonstrated that conserved signaling pathways are associated with the coordination of biological changes related to appressorial development and maturation. However, the downstream molecular pathways that are activated in developing appressoria remain mostly uncharacterized. Filling this gap would require a specific aim at characterizing tissue-specific regulators. Signaling pathways often converge on transcriptional regulation during cell development. Interestingly, the *MoHOX2* and *MoHOX7* genes were significantly downregulated in the two signaling-defective mutants *Δmac1* and *ΔMoplc1* [12],[17]. Given that both adenylate cyclase and phospholipase C are associated with the generation of signaling molecules, such as cAMP and Ca^2+^, it is possible that cAMP and/or Ca^2+^-dependent signaling pathways are involved in modulating the functions these two *MoHOX* genes.

As most homeobox TFs in other species are constitutively expressed [54], it is not surprising that *MoHOX2* and *MoHOX7* were constitutively expressed during development, but most highly expressed during conidiation and appressorium formation, respectively. Many TFs are also post-translationally regulated, especially by (de)phosphorylation [55],[56]. This event typically occurs in response to external stimuli, which lead to the modulation of the DNA-binding activity of homeobox TFs. This suggests that the two MoHOX proteins are phosphorylated or dephosphorylated by a kinase or phosphatase, respectively. The identification of such regulators responsible for (de)phosphorylating MoHOX proteins is in progress using a yeast two-hybrid system. Previously, a yeast two-hybrid study showed that PMK1 interacts with MST12 (MoHOX8), suggesting that PMK1 regulates MST12 to control invasive growth [30],[57]. PMK1 is a well-known MAP kinase essential for appressorium formation and invasive growth in *M. oryzae* [6]. Since MST12 is not involved in appressorium formation, there may be other TFs regulated by PMK1 that are involved in appressorium formation. Considering that MoHOX7 is another homeobox TF that is crucial for appressorium formation, PMK1 might interact with MoHOX7 for the regulation of appressorium development.

In summary, we have demonstrated that members of the homeobox TF family function as stage-specific regulators during *M. oryzae* development and pathogenicity. *MoHOX1*, *-2*, *-4*, *-6*, *-7*, and *-8* are specifically associated with hyphal growth and pigmentation, asexual reproduction, conidial morphology, mycelial growth, appressorium development, and invasive growth, respectively. Detailed molecular and cytological analyses revealed that deletion of the *MoHOX2* gene entirely abolished asexual reproduction, while other stages, including conidiophore development appeared normal. Also, the data showed that *MoHOX7* is a key regulator, essential for appressorium development on both hyphal tips and conidial germ tubes. These results provide evidence that *M. oryzae* is able to cause foliar disease via hypha-driven appressoria, after sensing environmental cues. Our studies will help to unveil the regulatory mechanisms involved in conidiation and appressorium formation and contribute to development of novel strategies for rice blast control.

## Materials and Methods

### Fungal strains and culture conditions

*Magnaporthe oryzae* strain KJ201 was obtained from the Center for Fungal Genetic Resources (CFGR) and was used as the wild-type stain in this study. The strain and its transformants were routinely grown at 25°C under continuous fluorescent light on oatmeal (50 g oatmeal per liter) agar medium or V8 (4% V8 juice) agar medium. DNA and RNA were isolated from mycelia, which were grown in liquid complete medium (0.6% yeast extract, 0.6% tryptone, 1% sucrose) for 4 days. Conidia were obtained from 10-day cultures on oatmeal agar media by rubbing the mycelia with water followed by filtration through Miracloth (Calbiochem, San Diego, USA). Germinated conidia were obtained by placing drops of conidial suspension on hydrophobic surfaces for 3 h. Appressoria were obtained by holding germinated conidia for an additional 3 h. For the phenotype assay, complete medium was used to measure the vegetative growth and colony characteristics [58]. Oatmeal agar medium and V8 juice agar medium were used to measure conidiation and conidial morphology.

### Nucleic acid manipulation and quantitative RT–PCR

Genomic DNA was isolated using two different methods, depending on the experimental purpose. Genomic DNA for general experiments was isolated according to a standard method [59]. Genomic DNA for PCR screening of transformants was prepared using the quick and safe method [60]. Restriction enzyme digestion, agarose gel separation, and DNA gel blotting were performed following standard procedures [59]. DNA hybridization probes were labeled with ^32^P using the Rediprime II Random Prime Labeling System kit (Amersham Pharmacia Biotech, Piscataway, NJ, USA) according to the manufacturer's instructions. The hybridization membrane was exposed to a Phosphorimager (BAS-2040, Fuji Photo Film, Tokyo, Japan) and visualized with the Phosphorimager software. Total RNA was isolated from frozen fungal mycelia using the Easy-Spin total RNA extraction kit (Intron Biotechnology, Seongnam, Korea) following the manufacturer's instructions. To measure the relative abundance of *MoHOX2* and *MoHOX7* transcripts in mutant backgrounds listed in Table 1, RNAs of the mutants were extracted from mycelia grown in CM liquid medium for 4 days at 25°C in a 120-rpm orbital shaker. The primer sets used to detect transcripts of conidiogenesis-related genes from *M. oryzae*, *A. nidulans*, and *N. crassa*, sets of primers are listed in Table 3 and Table S2.

### RNA isolation, RT–PCR, and gene expression analysis

For RT-PCR and quantitative real time RT-PCR (qRT-PCR), 5 µg of total RNA were reverse transcribed into first-strand cDNA using the oligo (dT) primer with the ImProm-II Reverse Transcription System kit (Promega, Madison, WI, USA) according to manufacturer's instructions. For detecting transcripts of the two complements, RT-PCR was conducted with primer pairs MoHOX2_ORF_F/MoHOX2_ORF_R and MoHOX7_ORF_F2/MoHOX2_ORF_R (Table S2). RT-PCR was performed in 20-µl reaction mixtures containing 100 ng cDNA, 2.5mM of dNTP mix, 2 µl 10×PCR buffer, 1 µl (10 pmol) of each primer, and 1 unit of *Taq* polymerase. In all, 30 cycles of RT-PCR were run on a Perkin-Elmer 9720 DNA thermal cycler. The β-tubulin gene was included as a control.

Real-time quantitative reverse transcription PCR (qRT-PCR) reactions were performed following previously established procedures [61]. The AB7500 Real-Time PCR system (Applied Biosystems, Foster city, CA, USA) was used for PCRs that consisted of 3 min at 95°C (1 cycle) followed by 15 s at 95°C, 30 s at 60°C, and 30 s at 72°C (40 cycles). Each qRT-PCR mixture (final volume 10 µl) contained 5 µl of Power SYBR® Green PCR Master Mix (Applied Biosystems), 3 µl of forward and reverse primers (100 nM concentrations for each) and 2 µl of cDNA template (12.5 ng/µl). The oligonucleotide sequences used for each gene are listed in Table S2. To compare the relative abundance of target gene transcripts, the average threshold cycle (Ct) was normalized to that of β-tubulin (MGG00604) for each of the treated samples as 2^−ΔCt^, where −ΔCt = (C_t, target gene_−C_t, β-tubulin_). Fold changes during fungal development and infectious growth in liquid CM were calculated as 2^−ΔΔCt^, where −ΔΔCt = (C_t, target gene_−C_t, β-tubulin_)_test condition_−(C_t, WT_−C_t, β-tubulin_)_CM_ [10]. qRT-PCR was performed with three independent pools of tissues in two sets of experimental replicates.

### Characterization of *MoHOX* deletion mutants

Vegetative growth was measured on complete agar medium on 10 days after inoculation, with three replicates. The ability to produce conidia was measured by counting the number of conidia from 6-day-old V8 juice agar plates as described previously [7]. Conidia were collected by flooding the plate with 5 ml of sterilized distilled water. The number of conidia was counted using a hemacytometer under a microscope. Conidiophore development was monitored as previously described [23]. Conidial size was measured as width by length under a microscope. Conidial germination and appressorium formation were measured on a hydrophobic coverslip. Conidia were harvested from 10-day-old oatmeal agar culture plates using sterilized distilled water. A conidial suspension of 40 µl was dropped onto a coverslip following adjustment of its concentration to approximately 5×10^4^ spores/ml. Drops were placed in a moistened box and incubated at 25°C. After 9 h of incubation, the percentage of conidia germinating and germinated conidia-forming appressoria was determined by microscopic examination of at least 100 conidia per replicate in at least three independent experiments, with three replicates per experiment.

### Plant infection assays

Plant penetration assays were performed using onion epidermis and rice sheaths, as previously described [62]. These experiments were replicated three times. For the pathogenicity assay, conidia were harvested from 8 to 10-day-old cultures on oatmeal agar plates and 10 ml of conidial suspension (10^5^ conidia/ml) containing 250 ppm Tween 20 were sprayed onto susceptible rice seedlings (*Oryza sativa* cv. Nakdongbyeo) at the three- to four-leaf stage. The inoculated plants were kept in a dew chamber at 25°C for 24 h in darkness and moved to a growth chamber with a photoperiod of 16 h with fluorescent lights. Disease severity was measured at 7 days after inoculation, as previously described [63]. For the infiltration infection assay, 100 µl of conidial suspension were injected into three points per leaf of 4-week-old rice plants. These experiments were replicated three times.

### Identification of homeobox transcription factors

Homeobox TFs were identified using InterPro terms (IPR001356 and IPR003120) for homeodomains via the pipeline of Fungal Transcription Factor Database (http://ftfd.snu.ac.kr/) [64].

### Phylogenetic analysis

Amino acid sequences of homeobox TFs were aligned using CLUSTAL W 1.83 [65]. Phylogenetic trees were constructed using the neighbor-joining method [66] with the aid of FTFD [64]. All sequence alignments were tested with a bootstrap method using 10,000 repetitions. The domain architecture of homeobox TFs was determined by InterProScan [67] and presented using the CFGP (http://cfgp.snu.ac.kr/) [68].

## Supporting Information

Figure S1 Generation of deletion mutants for *MoHOX* genes. The split-marker deletion method [Catlett, et al] and double-joint PCR system [Yu, et al] were applied to delete homeobox genes. Fragments corresponding to approximately 1.5 kb upstream and downstream of target genes were amplified with primers UF/UR and DF/DR (Table S2), respectively. A 2.1 kb the hygromycin phosphotransferase gene (HPH) cassette was amplified with primers HPHF and HPHR (Table S2) from pBCATPH [Kim, et al], which contains HPH [Gritz, et al]. The *MoHOX1* gene replacement constructs were amplified with primers UF/SplitDR and DR/SplitUF (Table S2) and the rest homeobox gene replacement constructs were used UF/DR primers, using fused products as a template. Protoplasts from KJ201 strain were directly transformed with purified PCR products of deletion construct fragments. Hygromycin-resistant transformants were selected on TB3 (0.3% yeast extract, 0.3% casamino acid, 1% glucose, 20% sucrose) media were supplemented with 200 µg/ml hygromycin B (Calbiochem, San Diego, CA, USA), and screened by PCR with primers SF/HPH1F or ORF_F/ORF_R (Table S2). Knock-out mutants were confirmed by southern blot hybridization. Briefly, genomic DNAs were digested with a restriction enzyme and hybridized with a probe, which are indicated on each schematic map (Figure S1). Molecular size of hybridized bands in the wild-type and transformants was compared to determine homology-dependent gene replacement events. Disruption of a targeted gene expression was reconfirmed in *ΔMohox2* and *ΔMohox7* mutants by RT–PCR analysis. For the complementation of the *ΔMohox2* and *ΔMohox7* mutant, a fragment amplified using UF/DR primers from the KJ201 genomic DNA was co-transformed with a geneticin resistance gene fragment into protoplasts of the *ΔMohox2* or *ΔMohox7* mutant (Table S2). Putative complemented transformants were selected on TB3 plates supplemented with 200 µg/ml of hygromycin B and geneticin G418 (Sigma Chemical Co., St. Louis, MO, USA), and tested for the integration and transcriptional expression of the *MoHOX* genes by DNA blot and RT–PCR. (Catlett NL, Lee BN, Yoder OC, Turgeon BG (2003) Split-marker recombination for efficient targeted deletion of fungal genes. Fungal Genet Newsl 50: 9–11.) (Yu JH, Hamari Z, Han KH, Seo JA, Reyes-Dominguez Y, et al. (2004) Double-joint PCR: a PCR-based molecular tool for gene manipulations in filamentous fungi. Fungal Genet Biol 41: 973–981.) (Kim S, Ahn IP, Rho HS, Lee YH (2005) MHP1, a Magnaporthe grisea hydrophobin gene, is required for fungal development and plant colonization. Mol Microbiol 57: 1224–1237.) (Gritz L, Davies J (1983) Plasmid-encoded hygromycin B resistance: the sequence of hygromycin B phosphotransferase gene and its expression in Escherichia coli and Saccharomyces cerevisiae. Gene 25: 179–188.)
(0.39 MB PDF)

Figure S2 Comparison of growth morphology of *ΔMohox1* on culture plates. Pictures were taken 5 days after inoculation of agar plugs (6 mm in diameter) on V8 juice agar plates. Abnormal increase in pigmentation and reduction in vegetative growth are obviously observed in the *ΔMohox1* mutant, compared to its wild-type and ectopic transformant.
(0.61 MB PDF)

Figure S3 Microscopic observation of conidial morphology of *ΔMohox4*. Conidia were harvested from 6-day-old V8 juice agar plates. Note that *MoHOX4* deletion reduced conidium size. Bars = 10 µm. Conidium width and length of *ΔMohox4* mutants were significantly smaller than those of the wild-type and *MoHOX4e* transformants (Table 2).
(0.33 MB PDF)

Figure S4 Comparison of growth morphology of *ΔMohox2* on culture plates. Pictures were taken 7 days after inoculation of agar plugs (6 mm in diameter) on oatmeal agar plates. No phenotypic difference was found on culture plates in comparison of the *ΔMohox2* mutant with its wild-type.
(0.46 MB PDF)

Table S1 Homeobox transcription factors in genomes of eukaryotic microbes.
(0.14 MB PDF)

Table S2 Oligonucleotides used in this study.
(0.12 MB PDF)

Table S3 Effect of the pharmacological signaling molecules on appressorium formation.
(0.11 MB PDF)
