# Supplementary figures and images for: Homeobox Transcription Factors Are Required for Conidiation and Appressorium Development in the Rice Blast Fungus Magnaporthe oryzae
Source: PLoS Genet. 2009 Dec 4;5(12):e1000757. doi: 10.1371/journal.pgen.1000757 (PMC2779367; doi:10.1371/journal.pgen.1000757)

Figure S1

**A**

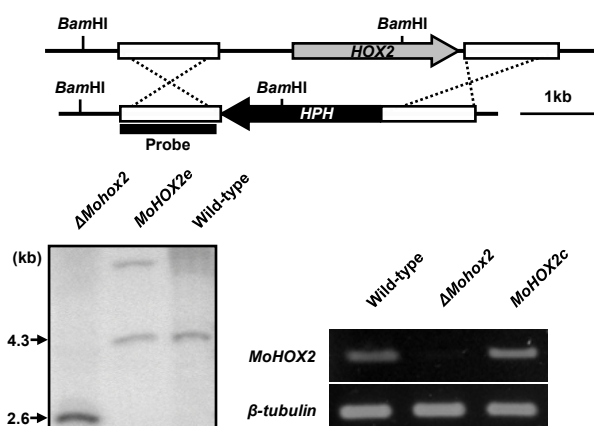

**B**

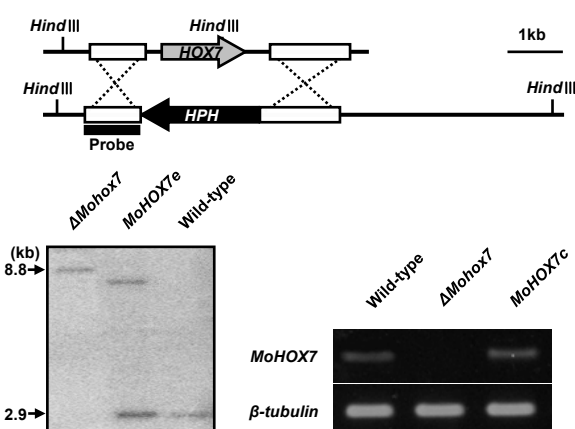

**C**

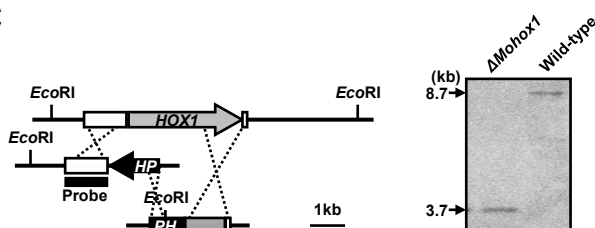

**D**

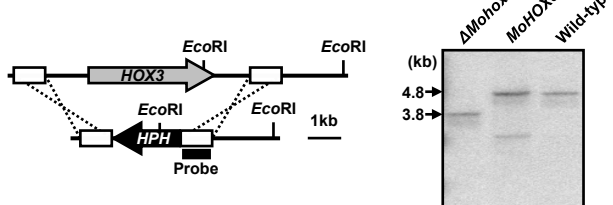

**E**

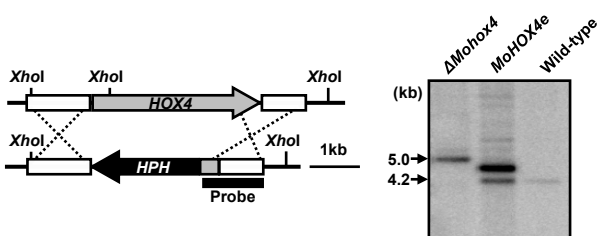

**F**

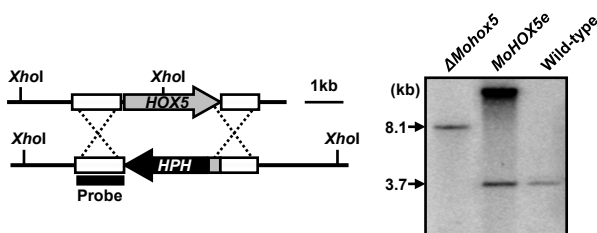

**G**

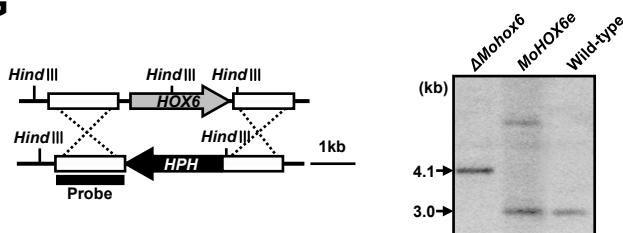

**H**

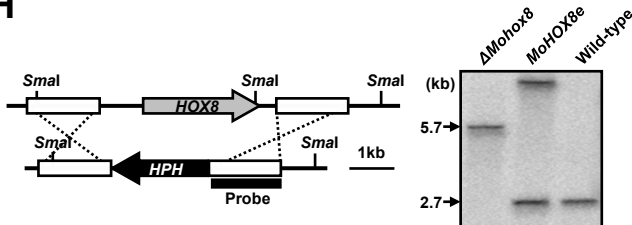

Supplement: Figure S1 — Generation of deletion mutants for MoHOX genes. The split-marker deletion method [Catlett, et al] and double-joint PCR system [Yu, et al] were applied to delete homeobox genes. Fragments corresponding to approximately 1.5 kb upstream and downstream of target genes were amplified with primers UF/UR and DF/DR (Table S2), respectively. A 2.1 kb the hygromycin phosphotransferase gene (HPH) cassette was amplified with primers HPHF and HPHR (Table S2) from pBCATPH [Kim, et al], which contains HPH [Gritz, et al]. The MoHOX1 gene replacement constructs were amplified with primers UF/SplitDR and DR/SplitUF (Table S2) and the rest homeobox gene replacement constructs were used UF/DR primers, using fused products as a template. Protoplasts from KJ201 strain were directly transformed with purified PCR products of deletion construct fragments. Hygromycin-resistant transformants were selected on TB3 (0.3% yeast extract, 0.3% casamino acid, 1% glucose, 20% sucrose) media were supplemented with 200 µg/ml hygromycin B (Calbiochem, San Diego, CA, USA), and screened by PCR with primers SF/HPH1F or ORF_F/ORF_R (Table S2). Knock-out mutants were confirmed by southern blot hybridization. Briefly, genomic DNAs were digested with a restriction enzyme and hybridized with a probe, which are indicated on each schematic map (Figure S1). Molecular size of hybridized bands in the wild-type and transformants was compared to determine homology-dependent gene replacement events. Disruption of a targeted gene expression was reconfirmed in ΔMohox2 and ΔMohox7 mutants by RT–PCR analysis. For the complementation of the ΔMohox2 and ΔMohox7 mutant, a fragment amplified using UF/DR primers from the KJ201 genomic DNA was co-transformed with a geneticin resistance gene fragment into protoplasts of the ΔMohox2 or ΔMohox7 mutant (Table S2). Putative complemented transformants were selected on TB3 plates supplemented with 200 µg/ml of hygromycin B and geneticin G418 (Sigma Chemical Co., St. Louis, MO, USA), an [file pgen.1000757.s001.pdf]

Figure S2

**Wild-type**

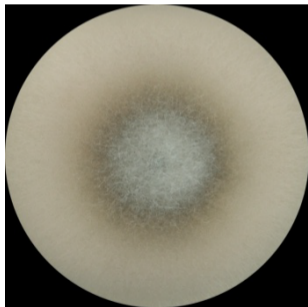

***ΔMohox1***

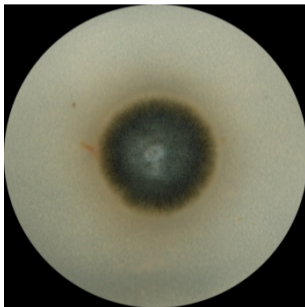

***MoHOX1e***

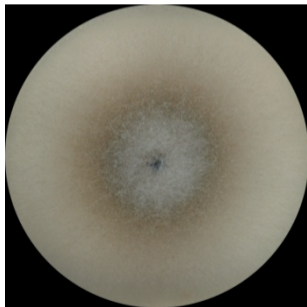

Supplement: Figure S2 — Comparison of growth morphology of ΔMohox1 on culture plates. Pictures were taken 5 days after inoculation of agar plugs (6 mm in diameter) on V8 juice agar plates. Abnormal increase in pigmentation and reduction in vegetative growth are obviously observed in the ΔMohox1 mutant, compared to its wild-type and ectopic transformant. (0.61 MB PDF) [file pgen.1000757.s002.pdf]

Figure S3

**Wild-type**

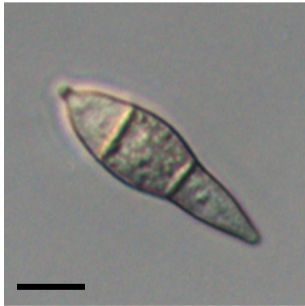

***ΔMohox4***

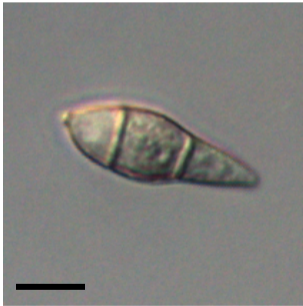

***MoHOX4e***

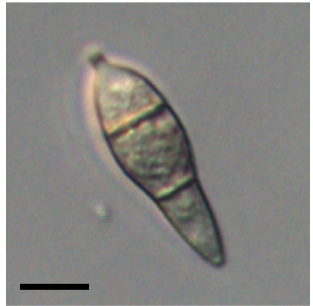

Supplement: Figure S3 — Microscopic observation of conidial morphology of ΔMohox4. Conidia were harvested from 6-day-old V8 juice agar plates. Note that MoHOX4 deletion reduced conidium size. Bars = 10 µm. Conidium width and length of ΔMohox4 mutants were significantly smaller than those of the wild-type and MoHOX4e transformants (Table 2). (0.33 MB PDF) [file pgen.1000757.s003.pdf]

Figure S4

**Wild-type**

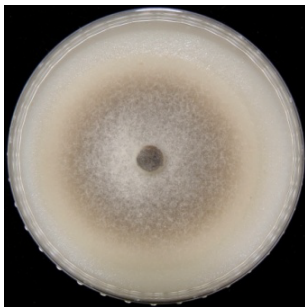

***ΔMohox2***

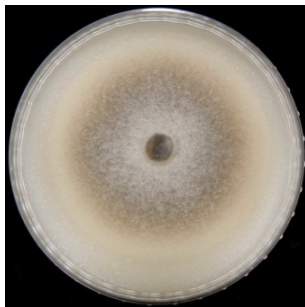

Supplement: Figure S4 — Comparison of growth morphology of ΔMohox2 on culture plates. Pictures were taken 7 days after inoculation of agar plugs (6 mm in diameter) on oatmeal agar plates. No phenotypic difference was found on culture plates in comparison of the ΔMohox2 mutant with its wild-type. (0.46 MB PDF) [file pgen.1000757.s004.pdf]
