# Supplementary material for: Homeobox Transcription Factors Are Required for Conidiation and Appressorium Development in the Rice Blast Fungus Magnaporthe oryzae
Source: PLoS Genet. 2009 Dec 4;5(12):e1000757. doi: 10.1371/journal.pgen.1000757 (PMC2779367; doi:10.1371/journal.pgen.1000757)
Supplement: Table S1 — Homeobox transcription factors in genomes of eukaryotic microbes. (0.14 MB PDF) [file pgen.1000757.s005.pdf]

Table S1. Homeobox transcription factors in genomes of eukaryotic microbes.

Species name	Phylum /Subphylum	Expected number			Homeobox TF /TF (%)	References
		ORFs	TFs	Homeobox TFs		
Magnaporthe oryzae	Pezizomycotina	11,069	462	8	1.73%	(Dean et al., 2005)
Aspergillus fumigatus	〃	9,887	562	9	1.60%	(Nierman et al., 2005)
A. nidulans	〃	10,701	638	7	1.10%	(Galagan et al., 2005)
Fusarium graminearum	〃	13,321	660	13	1.97%	(Cuomo et al., 2007)
F. oxysporum	〃	17,608	814	13	1.60%	-
F. verticillioides	〃	14,199	629	10	1.59%	-
F. solani	〃	15,707	992	22	2.22%	-
Neurospora crassa	〃	9,842	420	8	1.90%	(Galagan et al., 2003)
Podospora anserina	〃	10,596	477	7	1.47%	(Espagne et al., 2008)
Stagonospora nodorum	〃	16,597	516	13	2.52%	(Hane et al., 2007)
Trichoderma reesei	〃	9,129	513	8	1.56%	(Martinez et al., 2008)
Candida albicans	Saccharomycotina	6,090	308	5	1.62%	(Jones et al., 2004)<br>(van het Hoog et al., 2007)
C. glabrata	〃	5,165	262	10	3.82%	(Dujon et al., 2004)
Saccharomyces cerevisiae	〃	6,692	295	11	3.73%	(Goffeau et al., 1996)
Schizosaccharomyces pombe	Taphrinomycotina	5,058	223	2	0.90%	(Wood et al., 2002)
Cryptococcus neoformans	Basidiomycota	6,475	307	9	2.93%	(Loftus et al., 2005)
Laccaria bicolor	〃	20,614	501	15	2.99%	(Martin et al., 2008)
Phanerochaete chrysosporium	〃	10,048	387	11	2.84%	(Martinez et al., 2004)
Ustilago maydis	〃	6,689	332	7	2.11%	(Kamper et al., 2006)
Encephalitozoon cuniculi	Microsprodia	1,996	108	13	12.04%	(Katinka et al., 2001)
Phytophthora ramorum	Oomycetes	15,743	641	8	1.25%	(Tyler et al., 2006)
P. sojae	〃	19,027	919	7	0.76%	(Tyler et al., 2006)
**Total**		**242,253**	**10,966**	**216**	**1.97%**	
