# Supplementary material for: Homeobox Transcription Factors Are Required for Conidiation and Appressorium Development in the Rice Blast Fungus Magnaporthe oryzae
Source: PLoS Genet. 2009 Dec 4;5(12):e1000757. doi: 10.1371/journal.pgen.1000757 (PMC2779367; doi:10.1371/journal.pgen.1000757)
Supplement: Table S2 — Oligonucleotides used in this study. (0.12 MB PDF) [file pgen.1000757.s006.pdf]

Table S2. Oligonucleotides used in this study.

Name	Sequences (5' → 3')
MoHOX1	
UF	CTTGGGTTCTTTAGCATGGC
UR	TAGGTGTCCCCAAGTTCCTG
DF	AAAGCGTCGAGCCAATTAAA
DR	CTCGTTTCCATGGTTTGCTT
ORF_F	GGTTTTCCCGGTGCCTTCC
ORF_R	GTCGCCGCCGTGATAAATGTC
SF	GGGTCGGGTTCCTAAAATGT
MoHOX2	
UF	CGAGTGGCTGCTCTTCTACC
UR	GGACGGGCTACTACCCTTTT
DF	TGCACAGCAACTGCTTTCTT
DR	AGTTTGGGAGTGTTTGACGC
ORF_F	AACCGGAGGGCAAAGGAAAAG
ORF_R	GTCATTGGCGAAAGCGAAGTGT
SF	CAGAAGGTGTGAAGCATGGA
Comple_UF	CTTGTCGCGGGCTAATGCTAA
Comple_DR	GCGGGGTGGCTGAATGTG
RTOF	TGGGGTTCTGCAGCCATGTT
RTOR	GTCCCGTGGTGTTACGTTCTGG
MoHOX3	
UF	ATTCCTGGTCATTATTTTACTGG
UR	AACGTCTTTACCGCACCTC
DF	TGAGGATGAGCGTCGCGAA
DR	TTCCACGGAGCTTGAACCCT
ORF_F	TGCGGCTTTTGCGGCTTAC
ORF_R	TTGCGGTTGTTGGTGAATCTTTG
SF	GGTTCAACGAAGCAAACGAT
MoHOX4	
UF	GAGCAAGTTGCCCAGGTAGA
UR	GGCTTGGACTGGATTGAAAA
DF	AGGATTGAGGCATCACAAGG
DR	ACACTGTTTTGGCTCACACG
ORF_F	GGGCCGGTCCACTTGTT
ORF_R	AGGGGGTCGCAGGTCTAAA
SF	CTGCTTCTTAGGTTCGCAGG

Table S2. Continued.

Name	Sequences (5’ → 3’)
MoHOX5	
UF	ACCTTGGTTCTCATTCCCCT
UR	CAGTCTCAGTCGACGAACCA
DF	CTACCGCTCACAGACGGAGT
DR	AAACAAAGCAAAATCGTGCC
ORF_F	ACCCGGTTTCTTATGAGCGAGTTT
ORF_R	GTGGCCCGTGGTAAGTGGAGTA
SF	ACCAAGAACCTGAGGCACAC
MoHOX6	
UF	CGTTGCCCAGTTGACATAGGT
UR	AGACCGGGAATACAGCATACG
DF	GCTGTCTTGAGTCTGGATGCC
DR	GACTCAACTGCGCTTCCTCG
ORF_F	CTTGCCGCGTATAGGAATGGAG
ORF_R	CGAAACGGGCGACTGAAGC
MoHOX7	
UF	TGCCCACATCCATAACCAACT
UR	GGCGGCGAATGATAAAATGTAG
DF	CGGGGGAGAGGACACAAT
DR	GGCGGGCAAACGACAACT
ORF_F	TTGCAGGGCCGTTGTATGTCTAAT
ORF_F2	CCCGCATGCTCCTTTCAAGAAG
ORF_R	GCGCTTGCGTTGCTTGTTGT
Comple_UF	AAAAGGCCCGCACTGTCG
Comple_DR	TCTCCCCCGCTCGTCTGA
RTOF	CGGACGGCTCCAAGATTCTCC
RTOR	CTGCCACGCTTCATGCCAA
MoHOX8	
UF	TTGATGATCGCTCGCCGC
UR	AGATTGTGGCTGCTGGACCG
DF	CAAAAGCATAGGGCGGAAACA
DR	GCCAAGTATGCACACCTGATC
ORF_F	CCGCGCCTTCTTTCGTGTC
ORF_R	CTGCGGGGGTTTTTGGTTTC

Table S2. Continued.

Name	Sequences (5’ → 3’)
Hygromycin phosphotransferase	
HPHF	CGACAGAAGATGATATTGAAGG
HPHR	CTCTAAACAAGTGTACCTGTGC
HPH1F	TCAGCTTCGATGTAGGAGGG
SplitDR	CGTTATGTTTATCGGCACTTTGC
SplitUF	GCTGCTCCATACAAGCCAACC
β-Tubulin	
TubF	TCGACAGCAATGGAGTTTACAAC
TubR	AGCACCAGACTGACCGAAGAC
TubF2	CTCCAGGGTTTCCAGATCAC
TubR2	CCTCACCAGTGTACCAATGC
Conidiogenesis related gene	
COS1_RTOF	CTCAGCCCACATACAACTACCAGC
COS1_RTOR	GACGACGATGATGATGGCGATG
CON7_RTOF	CCAGGCTGGTTCGGATGTATCTC
CON7_RTOR	CCTGGTTGGACCCTCCGT
ACR1_RTOF	CGACATGCTCAGCTCTTACAGG
ACR1_RTOR	TAGTCGTATCGCTCCGTGCCAT
MoSTUA_RTOF	GATGACCGTTCGAGCAGCAGTG
MoSTUA_RTOR	GCGGGGCGGTTCATTGTC
MoAPS1_RTOF	TAGCAACCATCAACGCAACGACC
MoAPS1_RTOR	GCACACGGCAAAGTGTCGTTGCGCTCAA
MoAPS2_RTOF	CGGCACGGGCGAGAAGA
MoAPS2_RTOR	TTTACCATGCCATCCGACACCT
MoFLBA_RTOF	AGCGCTGGATGGAGTCAAAGAG
MoFLBA_RTOR	TACGGCGTTTTGTGCATCCTC
MoFLBC_RTOF	GACCAAGTTCGCTCCAGACG
MoFLBC_RTOR	GTCCTCGGAGCCAGCCT
MoFLBD_RTOF	CCAGATCCATCAACGCCGC
MoFLBD_RTOR	TCAAGCACGGAAGCGACTCG
MoFLUG_RTOF	AGCTCGAGATTCCTTGCCCA
MoFLUG_RTOR	CAATCCGTTACAGCCTCATCCC
MoCON6_RTOF	CACAAGGCCAACCTCAA
MoCON6_RTOR	TCTCCATCTCCTCGAGAC
MoCON8_RTOF	GATTCATCCGCAGCAAA
MoCON8_RTOR	CACATCTTGCCAAACAGG
