# Supplementary material for: Homeobox Transcription Factors Are Required for Conidiation and Appressorium Development in the Rice Blast Fungus Magnaporthe oryzae
Source: PLoS Genet. 2009 Dec 4;5(12):e1000757. doi: 10.1371/journal.pgen.1000757 (PMC2779367; doi:10.1371/journal.pgen.1000757)
Supplement: Table S3 — Effect of the pharmacological signaling molecules on appressorium formation. (0.11 MB PDF) [file pgen.1000757.s007.pdf]

Table S3. Effect of the pharmacological signaling molecules on appressorium formation.

Surface	Strain	Appressorium formation (%)					
		No treatment	HDD (1 μM)	cAMP (10 mM)	DOG (20 μg/ml)	$CaCl_2$ (10 mM)	Treatment of all
Hydrophilic	Wild-type	2.6 ± 0.7	63.7 ± 4.3	43.7 ± 4.2	83.4 ± 7.7	5.8 ± 1.5	92.2 ± 1.1
	ΔMohox7	0.3 ± 0.5	0.9 ± 0.1	1.1 ± 0.5	0.6 ± 0.5	1.6 ± 0.5	3.0 ± 1.1
Hydrophobic	Wild-type	99.7 ± 0.5	98.8 ± 1.0	97.5 ± 0.8	97.9 ± 2.2	98.6 ± 0.5	98.8 ± 0.5
	ΔMohox7	0.3 ± 0.5	1.1 ± 0.5	0.9 ± 0.8	1.3 ± 0.6	0.6 ± 0.5	3.2 ± 1.3

Effects of chemicals on appressorium formation were investigated. Conidial suspension (10^5 conidia/ml) was placed on either the hydrophobic or hydrophilic side of cover slips, and mixed with following solutions to final concentrations: 10 mM cAMP (Sigma-aldrich, St. Louis, MO, USA), 1 μM 1,16-hexadecandiol (HDD, Sigma-aldrich), 20 μg/ml 1,2-dioctanoyl-*sn*-glycerol (DOG, Sigma-Aldrich), or 10 mM $CaCl_2 \cdot 2H_2O$. Appressorium formation was observed under a microscope 18 h after incubation.
